# Cadherin Expression and EMT: A Focus on Gliomas

**DOI:** 10.3390/biomedicines9101328

**Published:** 2021-09-26

**Authors:** Carolina Noronha, Ana Sofia Ribeiro, Ricardo Taipa, Diogo S. Castro, Joaquim Reis, Cláudia Faria, Joana Paredes

**Affiliations:** 1 Neurosurgery Department, Hospital de Santo António, Centro Hospitalar Universitario do Porto, 4099-001 Porto, Portugal; cnoronha@ipatimup.pt (C.N.); jlreis@icbas.up.pt (J.R.); 2 Cancer Metastasis Group, i3S—Instituto de Investigação e Inovação em Saúde, Universidade do Porto, 4200-135 Porto, Portugal; aribeiro@ipatimup.pt; 3 Faculty of Medicine, University of Porto, 4200-319 Porto, Portugal; 4 Neuropathology Unit, Hospital de Santo António, Centro Hospitalar Universitario do Porto, 4099-001 Porto, Portugal; rtaipa.neuropat@chporto.min-saude.pt; 5 Unit for Multidisciplinary Research in Biomedicine (UMIB), Institute of Biomedical Sciences Abel Salazar, University of Porto, 4050-313 Porto, Portugal; 6 Stem Cells & Neurogenesis Group, i3S—Instituto de Investigação e Inovação em Saúde, Universidade do Porto, 4200-135 Porto, Portugal; diogo.castro@i3s.up.pt; 7 Anatomy Department, Institute of Biomedical Sciences Abel Salazar, University of Porto, 4050-313 Porto, Portugal; 8 Neurosurgery Department, Hospital de Santa Maria, Centro Hospitalar Universitario Lisboa Norte, 1649-028 Lisboa, Portugal; claudiamfaria@gmail.com; 9 IMM—Instituto de Medicina Molecular Joao Lobo Antunes, Universidade de Lisboa, 1649-028 Lisboa, Portugal

**Keywords:** cadherins, EMT, gliomas

## Abstract

Cadherins are calcium-binding proteins with a pivotal role in cell adhesion and tissue homeostasis. The cadherin-dependent mechanisms of cell adhesion and migration are exploited by cancer cells, contributing to tumor invasiveness and dissemination. In particular, cadherin switch is a hallmark of epithelial to mesenchymal transition, a complex development process vastly described in the progression of most epithelial cancers. This is characterized by drastic changes in cell polarity, adhesion, and motility, which lead from an E-cadherin positive differentiated epithelial state into a dedifferentiated mesenchymal-like state, prone to metastization and defined by N-cadherin expression. Although vastly explored in epithelial cancers, how these mechanisms contribute to the pathogenesis of other non-epithelial tumor types is poorly understood. Herein, the current knowledge on cadherin expression in normal development in parallel to tumor pathogenesis is reviewed, focusing on epithelial to mesenchymal transition. Emphasis is taken in the unascertained cadherin expression in CNS tumors, particularly in gliomas, where the potential contribution of an epithelial-to-mesenchymal-like process to glioma genesis and how this may be associated with changes in cadherin expression is discussed.

## 1. Cadherins: The Main Regulators of Cell-Cell Adhesion

Cell-cell contact and adhesion are indispensable mechanisms for tissue-specific tasks and homeostasis by defining cell polarity and tissue compartmentalization [1,2]. Amongst the several families of adhesion molecules, the major one is the superfamily of cadherins (named for “calcium-dependent adhesion”), which are transmembrane proteins involved in the formation of adherens junctions (AJ) [3]. Cell-cell adhesion is mediated by extracellular cadherin domains, which function in a calcium-dependent way. In contrast, the intracellular cytoplasmic tail associates with numerous adaptor and signaling proteins collectively referred to as the cadherin adhesome [3]. The cadherin superfamily includes classical cadherins, protocadherins, desmogleins, desmocollins, and others [3], which structurally share extracellular cadherin repeats.

Classical cadherins include epithelial (E-), neuronal (N-), placental (P-), and retinal (R) cadherins, which were the first members of the superfamily to be identified (CDH1, CDH2, CDH3, and CDH4, respectively). They are calcium-binding proteins, characterized by an ectodomain containing five extracellular cadherin repeats, a transmembrane domain, and a cytoplasmic domain with highly conserved binding sites for p120-catenin and β-catenin [4]. The cytoplasmic domain also participates in cell-cell adhesion by stabilizing the cadherin/catenin complex at the membrane and binding this complex to the actin cytoskeleton. Moreover, the diversity of cadherin-binding molecules potentiates the crosstalk between cadherins and other cellular systems, pointing to their key role as regulators of cellular behavior. Indeed, although the cytoplasmic tails of different classical cadherins bind the same cytosolic proteins, and cadherin/catenin complexes appear to be similar in different cellular contexts, cadherin-mediated signaling is nevertheless highly dependent on the cellular context. It regulates a wide range of normal physiological processes, including embryo development, apoptosis, gene expression, cell proliferation, differentiation, and migration [5,6,7].

Cadherins assemble into strong adhesive intercellular junctions with subtype specificities, mainly through homophilic connections. Cells expressing a given cadherin are thought to preferentially adhere to those expressing the same cadherin subtype since the expression of distinct cadherins is involved in cell sorting [8]. However, cadherins’ expression in cells can also be heterogeneous, with cells expressing multiple cadherin subtypes, which leads to cadherin-mediated heterotypic adhesion [9,10]. Cadherin complexes are perceived as dynamic, undergoing cycles of assembly and disassembly [11,12]. It is the combination of qualitative and quantitative cadherin expression differences that most likely confers tissue-specific characteristics. These characteristics also include the number of shearing forces on cells [13], pointing to a biophysical basis for morphogenetic phenomena [14].

Cadherins play crucial roles during embryonic development and during the maintenance of adult tissues’ normal architecture [15,16]. Thus, several human diseases result from compromised cadherin expression and function, including skin, cardiovascular and neuronal disorders, and cancer, as recently reviewed by Vestweber and colleagues [17].

## 2. Cadherins Expression in Normal Adult Tissues

The paradigm for cadherin function stems mostly from studies concerning E-cadherin, with a vast amount of literature documenting E-cadherin’s impact on tissue architecture and morphogenesis [18,19]. Dynamic expression of E-cadherin was found to be a prerequisite for cell migration and morphogenesis during embryonic development [15]. A decrease in E-cadherin expression is observed, for instance, during gastrulation when mesoderm is formed [20] and in ectoderm during neurulation [21], while its re-expression is indispensable for skin or kidney organogenesis [17,18]. In many cases, dynamic E-cadherin expression takes place in the context of an Epithelial-to-Mesenchymal Transition (EMT), a developmental process characterized by loss of cell polarity and adhesion (characteristic of epithelial cells), with vast morphological changes and acquisition of cell motility (mesenchymal state) [18]. EMT is to a large extent coordinated by so-called classical EMT transcription factors: Snail, Slug, Twist, ZEB1, and ZEB2. These are well-characterized transcriptional repressors, all of which can directly bind to and repress E-cadherin promoter and other cell-cell adhesion and epithelial genes [22,23].

Less is known about the physiological roles of the other previously referred classical cadherins. N-cadherin has a broader expression profile, present in nervous, fibrous, and musculoskeletal tissues [24,25,26,27], and is considered a cell-cell adhesion molecule expressed by mesenchymal cells. Similar to E-cadherin, N-cadherin is critical in cell attachment [28], differentiation into specialized tissues [24,25,26,27], and influence signaling in various cellular processes, such as cell proliferation and apoptosis [29]. Although N- and E-cadherin share structural and functional characteristics, they usually show a mutually exclusive expression pattern during embryonic morphogenesis [30]. Notably, N-cadherin adhesion properties appear to be strongly tissue-specific. It mediates strong cell-cell adhesion in cardiomyocytes, but it is also expressed in migratory fibroblastic cells [24,31]. Concerning P-cadherin expression in human tissues, it partially overlaps with E-cadherin expression, possibly reflecting partial redundancy. The two proteins have high homology and differ mainly in their extracellular portions. P-cadherin is co-expressed with E-cadherin in embryonic stem cells and several adult epithelial tissues, including the breast, prostate, several organs of the digestive tract and urinary tract, lung, and endometrium (reviewed in [19,32]). Concerning R-cadherin in normal tissues, it is found to play an important role in brain segmentation and neuronal outgrowth and in lens, muscle, and kidney development.

### 2.1. Epithelial Tissues

Epithelia are robust tissues formed by sheets of cells organized as mono or multilayers that serve as effective barriers that support the structure and regulate functionally diverse organs, such as lung, gut, kidney, and epidermis (reviewed by [33]). Epithelia characteristically show strong cell-cell adhesion mediated by specialized adhesive sites, mainly tight junctions (TJ), adherens junctions (AJ), and desmosomes.

E-cadherin is expressed in all mammalian epithelia, being mainly co-expressed and located at the cell membrane and organizing the adherens junctions. Adherens junctions are located between the apical and basolateral membrane domains of epithelial cells and are linked to a circumferential actomyosin belt, a dynamic structure that participates in epithelial sheet remodeling and regulates epithelial tissue integrity [34,35,36]. Moreover, the interplay between E-cadherin complexes and the actin cytoskeleton enables resistance to cell deformation, granted by cell-cell adhesion; or otherwise triggers cellular remodeling, which tolerates epithelial plasticity [37,38].

Although a key component in epithelial polarization, E-cadherin is crucial to preserve epithelial tissue integrity and homeostasis through the stabilization of cell-cell interfaces and plays an important role in epithelial cell proliferation and migration control [39,40,41]. Accordingly, disruption of epithelial polarity has been shown to cause a wide range of human diseases [15,42,43,44]. Likewise, many studies have pointed to E-cadherin as a central protein in human epithelial cancers [45,46]. The loss of E-cadherin-mediated cell-cell adhesion is a prerequisite for tumor cell invasion. Reestablishing E-cadherin function in cultured tumor cells has been shown to reverse an invasive mesenchymal phenotype to a more benign and epithelial phenotype [45,47,48]. Furthermore, in several human cancer types, in parallel to E-cadherin loss, the de novo expression of mesenchymal cadherins, such as N-cadherin and cadherin-11, is observed during tumor progression [49,50,51]. These observations led to the concept of “cadherin switch” in cancer, parallel to what is observed during delamination and migration of epithelial cells during embryonic development.

P-cadherin expression is restricted to the basal layers of stratified epithelial tissues, including breast, skin, prostate, and lung [8,52,53]. When expressed, P-cadherin appears crucial for normal epithelial architecture [54] and cellular migration [55]. Furthermore, in addition to its role as an adhesion molecule, P-cadherin has been hypothesized to play an important role in cell differentiation and proliferation [56,57]. In the mouse mammary gland, P-cadherin knockout has been associated with abnormal mammopoiesis and increased risk for the development of preneoplastic lesions [58]. *CDH3*/P-cadherin mutations have been associated with abnormal development syndromes, including hypotrichosis with juvenile macular dystrophy (HJMD) and ectodermal dysplasia, ectrodactyly and macular dystrophy (EEM syndrome) [59,60].

### 2.2. Central Nervous System (CNS)

Cadherins are also highly expressed in the nervous system promoting cell-cell interactions within neural networks and dynamically contributing to neural development and function [61,62,63,64,65].

Various cadherins are widely expressed in neural tissues, including N-cadherin, cadherin-11, cadherin-6, cadherin-8, or M-cadherin [61,63,64,66]. Initially, E-cadherin is also expressed in embryonic ectoderm, being replaced by N-cadherin after neural induction, which leads to the formation of the neural plate [67]. As cell differentiation proceeds in the neural tube, cadherin expression becomes restricted to specific brain subdivisions. For instance, in the mouse developing brain, R-cadherin expression is distributed throughout the telencephalon and hindbrain, while cadherin-8 expression is restricted to the cerebellum and ventral thalamus [64].

In the adult brain, cadherins’ expression decreases and often remain localized to synapses or perisynaptic areas [65,68]. However, past observations suggest that cadherins are involved in various aspects of neural development and function, including organization of neuroepithelial layers [9,67], regulation of neuronal migration [69], complex functional subdivision, and early compartmentalization [70] by modulating region-specific adhesiveness. Furthermore, an important role in synaptic function and plasticity has been pointed out for cadherin-catenin complexes [65,68]. In particular, N-cadherin has been shown to play a similar role in neural tissues as E-cadherin in epithelia. This protein grants cell polarity and the maintenance, proliferation, and differentiation of neural progenitor cells [71].

In normal glial cells, N-cadherin is also thought to be the key cadherin. Although with scarce literature, it was identified in oligodendrocytes and their precursors [72], as well as in astrocytes [73,74]. Neural phenotypes associated with cadherin loss of function include defects in neural tube formation and cortical organization, cognitive and synaptic dysfunction, failure of sensory neurogenesis, CNS malformations, and craniofacial development. In addition, cadherin-catenin complexes are involved in CNS developmental steps and synapse formation and functions. It is thus not surprising that defects and alterations in the cadherin-catenin complex impair high-order neural functions. In particular, it has been associated with addiction-related phenotypes, schizophrenia, and bipolar disorder [75,76,77].

## 3. Cadherins Expression in Neoplastic Disease

### 3.1. Systemic Cancer

During oncogenesis in epithelial tissues, organized cell-cell adhesion and normal cell polarity are disrupted by genetic, epigenetic, and microenvironmental changes, leading to abnormal signaling, loss of contact inhibition, altered stromal interactions, and cell migration (reviewed in [19]). E-cadherin was investigated in seminal studies regarding its role in carcinomas, which are tumors of epithelial origin. Since then, various studies have proposed the CDH1/E-cadherin coding gene as a tumor suppressor gene by enabling complex mechanisms to promote tissue organization and apoptosis blockade [5,49,78]. These mechanisms include both biophysical cell-cell adhesion processes and intracellular signaling coupled to inhibition of proto-oncogenic molecules, such as βcatenin and epidermal growth factor receptor (EGFR) [18,79]. Moreover, suppression of E-cadherin function has been linked to increased cell migration and invasion and induction of a mesenchymal cellular morphology [80]. E-cadherin loss was demonstrated in a variety of cancers, including lobular breast carcinomas [78], gastric adenocarcinoma [81], hepatocellular carcinoma [82], melanoma [83], squamous cell carcinomas of the skin [84], as well as esophagus [85], and head and neck carcinomas [86,87]. More recently, data from other cancers have questioned the loss of E-cadherin expression as a ubiquitous marker of disease aggressiveness [88,89]. In later stages, invasive and poorly differentiated breast and ovarian carcinomas, for example, still express E-cadherin, which marks the existence of a mesenchymal to epithelial transition during tumor progression or a hybrid or mixed epithelial-mesenchymal phenotype [90,91]. Since the initial reports, the role of other cadherins in cancer, including its actions as synergists or antagonists with E-cadherin, has been sought. Importantly, the structural diversity of cadherins and, particularly, their tissue-specific expression profiles and actions were indicators that cadherins’ role in oncogenesis was most likely heterogeneous. Initial studies in carcinomas have labeled N-cadherin as a “mesenchymal” biomarker; and tied it to the end-stage of a pathological, cancer-related EMT [92]. Indeed, cadherins frequently exhibit a homotypic binding pattern across cells. For N-cadherin, frequently expressed in stromal and endothelial cells, this leads to epithelial heterotypic cell-cell adhesion, facilitating local invasion and dissemination of carcinoma cells [93]. Various studies have elicited the role of N-cadherin in tumor progression [94]; however, in others, N-cadherin loss has been linked to a worse prognosis [95]. As for E-cadherin and N-cadherin, P-cadherin cancer-related function seems to be context-dependent [96,97]. In breast cancer, expression of P-cadherin is characteristically observed in tumors with high proliferative rates and decreased cell differentiation, strongly associated with poor patient survival [57,98,99]. By contrast, in colon carcinomas, P-cadherin is strongly expressed in well-differentiated, while mostly absent in poorly differentiated colon tumors [100]. P-cadherin overexpression with E-cadherin suppression and N-cadherin induction are considered components of the cadherin switch found in cancer-associated EMT responsible for tumor differentiation and progression [101]. Nevertheless, rising evidence points to the importance of considering the cancer-dependent role of these proteins, as much as the putative interactions between them. In a model of breast cancer, co-expression of P- and E-cadherin was associated with aggressive high-grade breast carcinomas by P-cadherin-mediated disruption of functional cadherin-catenin complexes, thereby obstructing the E-cadherin tumor-suppressive role [97,102,103,104]. Considering R-cadherin impact on tumorigenesis, its role is still controversial. Conflicting data points R-cadherin as either promoting tumor progression [105] or inhibiting it [106,107].

### 3.2. CNS Tumors

As mentioned before, N-cadherin is the most commonly expressed cadherin in the CNS. E-cadherin expression, in particular, is rare and localized to arachnoid cells, choroid plexus, and anterior pituitary [108]. Peripheral nervous system cells, including Schwann cells, were also found to express E-cadherin. This protein plays an important role in cell adhesion and maintenance of peripheral nervous system architecture [109,110,111]. Consequently, the role of E-cadherin expression in tumors derived from these locations has been studied and described in meningiomas, choroid plexus papilloma, schwannomas, and pituitary adenomas [108,112,113,114,115,116].

#### 3.2.1. Pituitary Adenomas

Pituitary adenomas are epithelial tumors with neuroendocrine differentiation, immunohistochemically subdivided according to the presence of pituitary hormones and transcription factors. Variable E-cadherin and N-cadherin staining has been described in these tumors [114,117,118,119]. In particular, loss of E-cadherin expression has been linked to increased tumor dimensions and invasive behavior in growth hormone and prolactin-producing pituitary adenomas [114,120,121,122]. In addition, response to pharmacological treatment with somatostatin analog was worse for somatotroph adenoma without E-cadherin expression [114]. In a surgical series of 52 patients, E-cadherin loss and N-cadherin expression accompanied morphological changes suggestive of an EMT-like process, unique to a subset of invasive pituitary adenomas [123].

#### 3.2.2. Meningiomas

Various studies have found E-cadherin to be expressed in diverse subtypes of meningiomas, tumors derived from arachnoid cap cells [108,112,116]. Furthermore, E-cadherin downregulation has been described as one of the main molecular events responsible for meningioma development, and alterations within the CDH1 gene have been found in this neoplastic disease, namely loss of heterozygosity (LOH) and genomic instability [112,124]. Loss of E-cadherin expression has been associated with tumor proliferation, invasiveness, and dedifferentiation [116,125,126]. In addition, E-cadherin expression is less frequently observed in atypical (WHO grade II) meningiomas and is commonly absent in anaplastic meningiomas (WHO grade III), which are considered malignant [124,126]. Moreover, one study found that E-cadherin loss of expression was a significant predictor of tumor recurrence in meningioma patients [126].

In contrast to meningiomas and pituitary adenomas, the role of classical cadherins in other CNS tumors remains to be clarified, as published data remain limited and conflictive. For instance, leptomeningeal dissemination has been associated with increased N-cadherin expression in medulloblastoma [127], while lower expression levels were identified in disseminated neuroblastoma [95] and ependymoma [128]. Importantly, a recent study showed frequent positivity for N-cadherin in ependymoma and identified increased N-cadherin levels as a predictor of earlier tumor recurrence, arguing a contrary role for N-cadherin as a marker of worse prognosis in ependymoma [129]. Studies on E-cadherin expression show that this adhesion protein is mostly absent in neuro-epithelial tumors, such as ependymoma and medulloblastoma [113,125,127].

#### 3.2.3. Gliomas

The expression patterns of classical cadherins in gliomas have been evaluated by several, yet contradicting, studies. Gliomas characteristically diffusely infiltrate the underlying cerebral parenchyma, which has normal N-cadherin expression. This behavior reflects a higher tendency toward tumor invasion and migration, which rely on cell-cell adhesion mechanisms.

N-cadherin expression in gliomas has been described in various patient cohorts, with approximately 60–80% of positive cases in glioblastoma series [130,131]. However, these studies are discordant regarding the correlation between N-cadherin immunoreactivity with patient prognosis. In 1995, Shinoura et al. studied the differences in N-cadherin expression in the normal brain parenchyma and different gliomas, including pilocytic astrocytomas, low-grade oligodendrogliomas or astrocytomas, anaplastic astrocytomas, and glioblastomas. Although mRNA levels for N-cadherin were significantly higher in glioblastomas, protein expression was similar among high-grade and low-grade gliomas and normal brains. Moreover, no consistent association between invasiveness capacity and N-cadherin expression was found [132]. As for Shinoura et al., other studies have found discrepant results between N-cadherin mRNA and protein expression levels [133,134]. These results point to the importance of protein stability in the tumor microenvironment. Indeed, a study showed that N-cadherin cleavage by ADAM-10 occurred at a significantly higher rate in glioblastoma cells than in normal brain [135], and it has been further suggested that N-cadherin cleavage is a prerequisite for glioblastoma cell migration [5,135].

Other studies have supported a protective role for N-cadherin in gliomas. In glioma models, N-cadherin down-regulation has been associated with altered cell polarization and abnormal motile behavior [136], with a significant increase in tumor cell migration and invasive capacity [136,137]. In surgical specimens, expression levels evaluation pointed to a decrease in N-cadherin immunoreactivity upon glioblastoma recurrence [138,139] and association to tumor cerebrospinal fluid (CSF) dissemination [139]. In line with this, another report described how even redistribution of N-cadherin at the cell membrane is induced by cleaving its intracellular anchorage to the cytoskeleton by ROBO1, promoting invasiveness of glioblastoma cancer stem cells [140].

Nevertheless, contradictory results have questioned the role above of N-cadherin in glioma genesis. N-cadherin protein expression levels were also shown to increase according to pathological glioma grade [130,141] and correlate with Ki-67 labeling index [130], suggesting a role for cell adhesion signaling in tumor cell proliferation and dedifferentiation. Importantly, a trend towards decreased survival with increased expression of N-cadherin has also been described in different cohorts [131,141]. In a very recent publication, Gritsenko et al. studied the mechanisms of brain infiltration by glioma cells. They proposed an AJ-mediated mechanism for glioma migration and progression, dependent on N-cadherin and p120-catenin complex [142]. Similarly, Osuka et al. showed an N-cadherin expression increase among the population of radioresistant glioma stem cells (GSC), with increased stemness and reduced proliferation [143].

E-cadherin expression has been more commonly described as scarce or absent in gliomas [120,125,131,141] while contradicting results regarding its role in disease progression have been hypothesized. Motta et al. analyzed E-cadherin mRNA levels in 81 neuroepithelial tumors, including 62 astrocytic tumors and those from WHO grade I to IV. Low-grade astrocytomas (grades I-II) showed higher mRNA levels than did high-grade astrocytomas [144]. Likewise, Bar et al. reported E-cadherin staining to be more commonly positive in grade II (43%) over grade IV gliomas (23%) [145]. These results suggest that a decrease in E-cadherin gene expression underlies astrocytoma progression. Moreover, in a series of low-grade gliomas, CDH1 promoter methylation status was found to be frequent (65% astrocytomas, 66% oligodendrogliomas, and 57% oligoastrocytomas) and hypermethylation status associated with shorter progression-free survival [146]. In the same study, negative immunoreactivity for E-cadherin correlated with shorter patient progression-free and overall survival [146]. Despite these results, the assumption that E-cadherin plays the same protective role in gliomas as in most systemic cancers remains highly dubious. Opposing data documented E-cadherin expression to be higher in high-grade gliomas than in low-grade gliomas [147]. Furthermore, in particular subtypes of glioblastoma, with epithelial and pseudo-epithelial differentiation and high E-cadherin positivity, E-cadherin expression correlated with a worse prognosis [148,149]. In glioma cell models, E-cadherin expression was confirmed to be a rare event but to significantly influence in vitro growth and invasion capacity [148]. All this information is summarized in Table 1.

Almost inexistent literature can be found for the other two classical cadherins in glioma genesis. Nonetheless, contradicting data for R-cadherin has been described. On the one hand, R-cadherin increased expression has been shown at the cell-cell junctions after PDGF-B-induction in an experimental model of high-grade oligodendrogliomas [151], thus suggesting a role in glioma genesis. On the other side, it has been demonstrated that it is upregulated after autophagy induction, promoting a more epithelial and less aggressive phenotype of glioblastoma cell lines [152].

## 4. EMT and Cadherin Switch in Gliomas

Glioma capacity to grow, invade and resist conventional therapy, akin to systemic cancer, relies on EMT-like cellular programs [153]. However, the significance of the EMT and MET (mesenchymal to epithelial transition) programs in cancer progression and metastasis remains to be fully elucidated. In particular, how the concept of EMT applies to glioblastoma and other factors tumors with non-epithelial origin.

**Figure 1:**
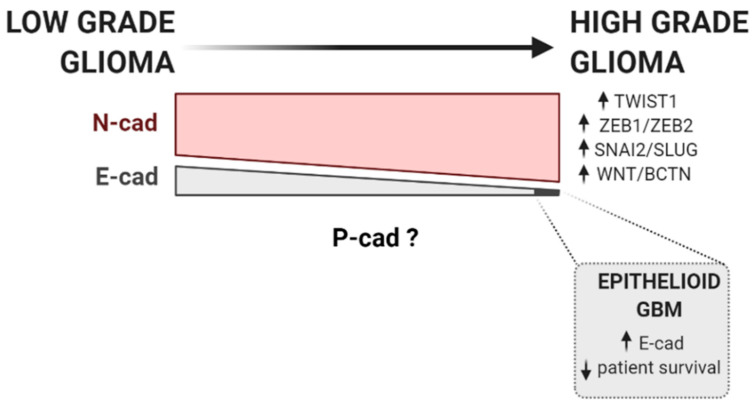
Cadherins and EMT factors expression during glioma progression. The progression of low-grade to high-grade glioma is commonly accompanied by an increase in the activity of classical EMT transcription factors and other well-described EMT-promoting pathways, such as ZEB1/ZEB2 [140,160,161], TWIST1 [164,165], WNT/β-catenin pathway [167], and SNAI2/SLUG [159,169]. According to current data available, N-cadherin is generally expressed in low-grade glioma, but its expression has been described to be increased in high-grade gliomas [130,141]. On the contrary, E-cadherin is fairly absent in gliomas. Still, its expression is more frequent in low-grade gliomas than in glioblastoma [144,145]. Notwithstanding, in aggressive glioblastoma subtypes, with epithelial and pseudo-epithelial differentiation, E-cadherin is found to be overexpressed [148]. Upward row represents increased expression, whereas downward row indicates decreased patient survival rate.

In 2010, transcriptomic analysis of glioblastoma established the presence of a molecular classification into four subtypes: proneural, neural, classical, and mesenchymal [154]. Since then, the mesenchymal subtype of glioblastoma has been thoroughly investigated and suggested to associate with an invasive phenotype, increased aggressiveness, and robust treatment resistance [153,155,156,157]. The mesenchymal phenotype in glioblastoma has, in addition, been correlated with worse overall survival [154]. Moreover, the mesenchymal transformation has been documented in glioblastoma upon tumor recurrence [155,156], as well as a response to various insults, including temozolomide [158] and radiation treatments [159]. Perhaps the strongest evidence for an EMT-like process in glioblastoma is the activity of classical EMT transcription factors and other well-described EMT-promoting pathways, such as ZEB1/ZEB2 [140,160,161,162,163], TWIST1 [164,165,166], WNT/β-catenin pathway [167,168] and SNAI2/SLUG [159,169] (Figure 1). An EMT-like program in gliomas is thus increasingly recognized and correlated to the phenomenon of glioblastoma progression and invasion. However, the canonical EMT-related E- to N-cadherin switch is unlikely to be an essential process. Increasing evidence suggests that cadherins may not be restrictive to have a specific role in tumorigenesis but instead can work as tumor suppressors or promotors depending on the tissue and tumor-associated context [170,171,172,173].

Taken together, published data point for a broad N-cadherin expression in glioblastomas, with a likely increase from low grade to high-grade gliomas [130,141]. The tumorigenic result of such expression is disputed, as it spans from decreased invasiveness and dissemination capacity [136,137,139] to a worse prognosis with decreased overall survival in clinical series [131,141] (Figure 1). Data on E-cadherin, whose expression is, on the contrary, fairly absent in gliomas, proposes E-cadherin loss as a potential step in glioma genesis, as the expression is more frequent in low-grade gliomas than in glioblastoma [144,145]. Notwithstanding, important contradictory data in apparent aggressive glioblastoma subtypes, with epithelial and pseudo-epithelial differentiation and high E-cadherin expression, questions such a proposed benign role for E-cadherin in gliomas [148] (Figure 1). It is nevertheless misleading to simply interpret these results in the context of a systemic EMT. They emerge from a collection of small sampled patient series [120,125,130,132,138,141,145,146,149], in which E-cadherin expression is substantially lower and N-cadherin expression considerably higher than in published carcinomas series [57,174,175,176,177]. Importantly, no key reciprocal or concomitant down and upregulation for these proteins was explored or suggested, as the role of cadherin co-expression was not explored. Moreover, data on P-cadherin expression in glioblastoma is at the moment inexistent Figure 1.

Caution is therefore mandatory when interpreting results for cadherin expression and its role as EMT biomarkers in glioblastoma, as proof of a cadherin switching process remains elusive in glioblastoma and cadherin expression contribution to glioma progression is still unclear.

## 5. Conclusions

Cadherins are key proteins in normal development, and tumor pathology and data on cadherin expression in gliomas point to a distinct pattern of expression when compared to classical epithelial cancers, in which the prognostic significance of each cadherin remains elusive. In order to understand how cadherin expression reflects EMT and differentiation status in gliomas, as well as propensity to invade and migrate, it is crucial to develop large-scale studies in low-grade to high-grade gliomas, with the concomitant evaluation of both epithelial and mesenchymal cadherins.

## Figures and Tables

**Table 1:** Summary of cadherin’s expression and prognosis in different published patient glioma series. NA (not assessed).

Author/Year	Cadherin and Method	Result	Prognosis
Howng et al. 2002 [120]	E-cadherin expression by RT-PCR in 16 astrocytomas and 29 other SNC tumors	Ecad staining in 2/16	NA
Schwechheimer et al. 1998 [125]	E-cadherin Immunohistochemistry of 42 gliomas (7 grade I astrocytoma, 6 grade II astrocytima, 5 grade II oligodendroglioma, 14 grade III astrocytoma, 8 GBM) and other 105 SNC tumors	No Ecad staining in gliomas	NA
Utsuki et al. 2004 [127]	E- and N-cadherin Immunohistochemistry of 45 gliomas (18 GBM, 16 grade III anaplastic astrocytomas, 11 grade II diffuse gliomas).	No Ecad staining Ncad staining in GBM (81%) and anaplastic astrocytoma (31%)-Ncad staining increases with WHO grade.	Ncad staining associated with a worse prognosis
Noh et al. 2017 [131]	E- and N-cadherin Immunohistochemistry of 92 gliomas	Ecad expression in 8.7% Ncad expression in 88.0% No significant difference in OS, PFS increased in low Ncad expression.	Tendency for worse prognosis with Ncad expression
Shinoura et al. 1995 [132]	N-cadherin mRNA and protein level in 21 gliomas (one pilocytic astrocytoma, 4 grade II astrocytomas, 3 grade III anaplastic astrocytoma, 9 GBM, 2 mixed gliomas, 2 grade II oligodendroglioma)	Ncad expression equal (protein) to higher (mRNA) in GBM	No prognosis
Asano et al. 1997 [138]	N-cadherin Immunohistochemistry of 22 astrocytomas (13 GBM and 9 anaplastic grade III astrocytoma)	No Ecad expression Ncad expression in all primary tumors. Ncad loss associated with tumor dissemination.	Ncad staining better prognosis.
Wu et al. 2013 [141]	E- and N-cadherin Immunohistochemistry of 40 brainstem gliomas (30 low grade and 10 high-grade) and RT-PCR of 18 brainstem gliomas (10 low grade and 8 high grade)	No Ecad staining, weak mRNA level. N-cad expression increased with WHO grade and showed a trend toward shorter survival.	Ncad expression associated with a worse prognosis
Motta et al. 2008 [144]	E-cadherin RT-PCR in 62 astrocytomas (21 grade I astrocytomas, 10 grade II astrocytomas, 10 grade III astrocytomas, and 21 GBM) and 19 other SNC tumors	Ecad expression is higher in low-grade astrocytomas than high-grade astrocytomas. Ecad expression decreases with WHO grade.	Ecad expression associated with a better prognosis
Bar et al. 2014 [145]	E-cadherin Immunohistochemistry of 92 gliomas (23 grade I pilocytic astrocytoma, 23 grade II astrocytoma, 7 grade II oligodendroglioma, and 39 GBM.	Ecad staining in 28.8% of gliomas, no differences with WHO grade.	No prognosis
D’Urso et al. 2011 [146]	E-cadherin Ecad gene (CDH1) promoter methylation and E-cad expression by methylation-specific polymerase chain reaction (MSP) and immunohistochemistry in 84 low-grade gliomas (43 diffuse astrocytomas, 27 oligodendrogliomas, and 14 oligoastrocytomas)	CDH1 promoter hypermethylation in 65% of astrocytomas, 66% oligodendrogliomas, and 57% oligoastrocytomas correlated with a worse prognosis. Ecad expression positive in 15/43 astrocytomas, 9/27 oligodendrogliomas, and 6/14 oligoastrocytomas. Loss of immunoreactivity for E-cadherin correlated with worse survival	Loss of Ecad expression worse prognosis
Lewis-Tuffin et al. 2010 [148]	E-cadherin Immunohistochemistry of TMAs of 83 cases of GBM or anaplastic astrocytoma and 31 gliomas and immunohistochemistry of 27 GBM with epithelial/pseudo-epithelial differentiation	No Ecad expression in TMA Ecad staining in 33% of GBM with epithelial/pseudo-epithelial differentiation. Worse overall survival in patients with Ecad expression.	Ecad staining associated with a worse prognosis.
Rodriguez et al. 2008 [149]	E-cadherin Immunohistochemistry of 58 GBM with adenoid, epithelioid, or true epithelial features	Ecad staining in 82% of GBM.	NA
Darweesh et al. 2016 [150]	N-cadherin Immunohistochemistry of 60 GBM	Ncad expression in 88.3% cases. Ncad expression associated with tumor histological variant-more common in cases of Gliosarcoma than in glioblastoma with oligodendroglioma component.	NA

## Data Availability

Not applicable.
